# Leg Length, Body Proportion, and Health: A Review with a Note on Beauty

**DOI:** 10.3390/ijerph7031047

**Published:** 2010-03-11

**Authors:** Barry Bogin, Maria Inês Varela-Silva

**Affiliations:** Health & Lifespan Research Centre, School of Sport, Exercise & Health Sciences, Loughborough University, Loughborough, Leicestershire LE11 3TU, UK; E-Mail: m.i.o.varela-silva@lboro.ac.uk

**Keywords:** leg length, body proportions, health, disease risk, beauty

## Abstract

Decomposing stature into its major components is proving to be a useful strategy to assess the antecedents of disease, morbidity and death in adulthood. Human leg length (femur + tibia), sitting height (trunk length + head length) and their proportions, for example, (leg length/stature), or the sitting height ratio (sitting height/stature × 100), among others) are associated with epidemiological risk for overweight (fatness), coronary heart disease, diabetes, liver dysfunction and certain cancers. There is also wide support for the use of relative leg length as an indicator of the quality of the environment for growth during infancy, childhood and the juvenile years of development. Human beings follow a cephalo-caudal gradient of growth, the pattern of growth common to all mammals. A special feature of the human pattern is that between birth and puberty the legs grow relatively faster than other post-cranial body segments. For groups of children and youth, short stature due to relatively short legs (*i.e.*, a high sitting height ratio) is generally a marker of an adverse environment. The development of human body proportions is the product of environmental x genomic interactions, although few if any specific genes are known. The HOXd and the short stature homeobox-containing gene (SHOX) are genomic regions that may be relevant to human body proportions. For example, one of the SHOX related disorders is Turner syndrome. However, research with non-pathological populations indicates that the environment is a more powerful force influencing leg length and body proportions than genes. Leg length and proportion are important in the perception of human beauty, which is often considered a sign of health and fertility.

## 1. Introduction

A dispassionate naturalist from another planet on a collecting mission to Earth might be satisfied with a sample of one or two specimens of *Homo sapiens* as representative of the species. Human observers of our species are not so easily contented. This is because from the anthropocentric perspective human beings display a variety of sizes, shapes, colors, temperaments and other phenotypic characteristics. Professional anthropologists, physicians, and others have debated the cause and significance of human phenotypic variation for centuries. Much of the historical discourse focused on concepts of “race” and some of the dispute centered on the human status of various living groups of people [1,2]. Serious proposals about the hierarchy of humanness appeared as recently as 1962 with the publication of *The Origin of Races* by Carleton Coon [3], Professor of Anthropology at the University of Pennsylvania. Coon divided living peoples of the world into five “races” based, in part, on body size and proportions. The Australian Aborigines (designated “Australoids” by Coon), have exceptionally long legs in proportion to stature, and African pygmies (“Congoids” in Coon’s taxonomy), have exceptionally short stature, long arms relative to leg length, and especially short lower legs. In Coon’s words, “Their manner of dwarfing verges in the achondroplastic…” [3, p. 653]. Moreover, Coon asserted that both “races” crossed the species threshold between *Homo erectus* and *H. sapiens* only in the last 10,000–50,000 years. In contrast, Coon proposed that ancient Europeans (called “Caucasoids” by Coon), had crossed the *H. sapiens* threshold about 200,000 years ago. According to Coon, the ancient Europeans were “normal” in size and shape, able to “…sit in any western European restaurant without arousing particular comment except for their table manners” [3, p. 582].

These claims of race-based human taxonomy, including Coon’s time thresholds for homo-sapienation, have been discredited by paleontological and genomic research showing the antiquity of modern human origins within Africa, as well as the essential genomic African nature of all living human beings [4–6]. Coon’s claim that African pygmies have “achondroplastic proportions” is also wrong. Shea and Bailey [7] show that African pygmies are reduced in overall size and have a body shape that is allometrically proportional to the size reduction.

Discarding the racist history of the study of human morphology allowed research to focus on more meaningful biological, medical, social, and aesthetic implications of human body size and shape. In this article, we review the evidence that human body shape, especially the length of the legs relative to total stature, is an important indicator for epidemiology and environmental public health. We find that across the human species, as well as within geographic, social, and ethnic groups of people, relative leg length reflects nutritional status and health during the years of physical growth and also has biologically and statistically significant associations with risks for morbidity and mortality in adulthood.

## 2. Leg Length Defined

A strict anatomical definition of leg length (LL) is the length of the femur + tibia. Due to the bipedal nature of the human species, “leg length” often is measured as: (femur + tibia + the height of the foot, from the tibia-talus articulation to the ground). Alternatively, the phrase “lower limb length” may be use to denote this linear dimension. In this paper, we use “leg length” to denote any of the measurements described below in section 3. We do so because in a living human being it is difficult to measure anatomical LL. The maximum length of the femur is measured from its head, at the proximal end, to its medial condyle, at the distal end. In life, the femur and pelvic bones overlap and the head of the femur is difficult to assess due to its articulation within the acetabulum. A high degree of body fatness may make these bony landmarks difficult, or, impossible, to access. Consequently, LL is often defined by an easier to measure dimension such as iliac height (IH) and subischial leg length (SLL). It is also possible to measure an estimate LL via the combination of thigh length (TL) and knee height (KH). Some studies employ only one of these measures as the indicator of LL.

Each of these measurements can be transformed in ratios, generally in relation to total stature and sitting height (SH) to give indications of body proportions. In this article we discuss the sitting height ratio (SHR), relative subischial leg length (RSLL), and the knee height ratio (KHR).

## 3. Practical Methods and Techniques

Here we present a brief description of the anthropometric methods required to obtain various measures of leg length. More detail of the methods may be found in [8] and the NHANES anthropometric manual (http://www.cdc.gov/nchs/data/nhanes/nhanes3/cdrom/NCHS/MANUALS/ANTHRO.PDF). Our purpose in providing these descriptions is to show the variety of methods employed to estimate leg length, biases which may be associated with each method, the limits of comparability between methods, and the variety of anatomical growth centers and different biological growth processes that underlie the concept of “leg length.”

### 3.1. Iliac Height (IH)

**Figure 1.**
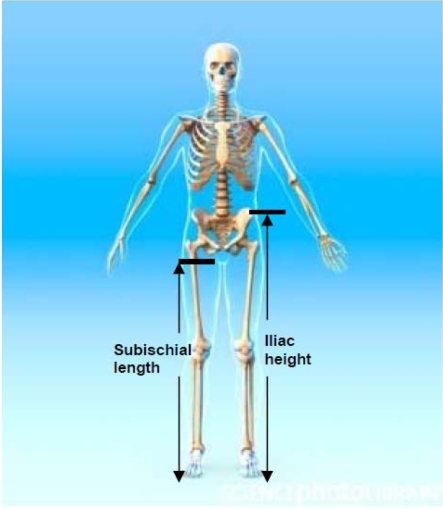
Iliac height and subischial length. Credit: Roger Harris/SCIENCE PHOTO LIBRARY, royalty free image, labelling added by the authors.

The distance between the summit of the iliac crest and the floor (see Figure 1).

### 3.2. Subischial Leg Length (SLL)

The difference between stature and sitting height. It assumes that in a seated position the proximal landmark corresponds to the hip joint, which is very difficult to locate (see Figure 1).

### 3.3. Thigh Length (TL)

**Figure 2.**
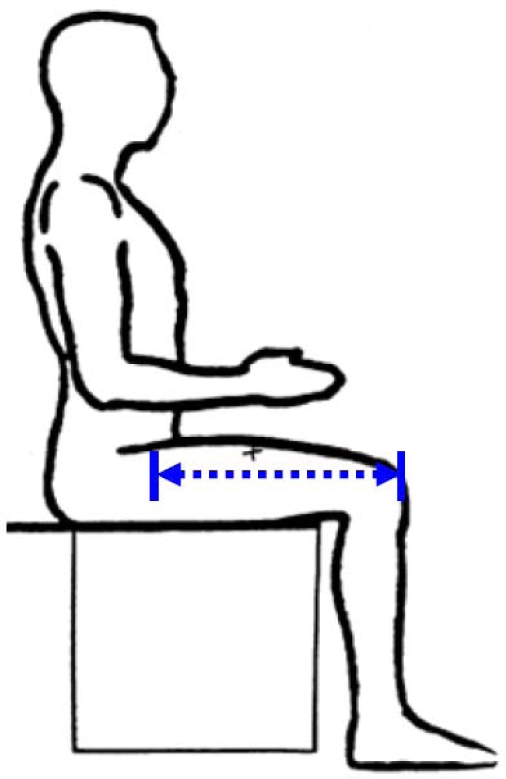
Thigh length (from NHANES anthropometric manual).

The distance between the proximal end of the greater trochanter and the distal lateral femoral condyle. Because in living humans it is difficult to locate these joints, TL is measured from the midpoint of the inguinal ligament to the proximal edge of the patella (see Figure 2). In overweight or obese people with excessive abdominal subcutaneous fat it may be difficult to find the inguinal ligament. Moreover, social and ethical prohibitions may prevent access to the site of the inguinal ligament.

### 3.4. Knee Height (KH)

**Figure 3.**
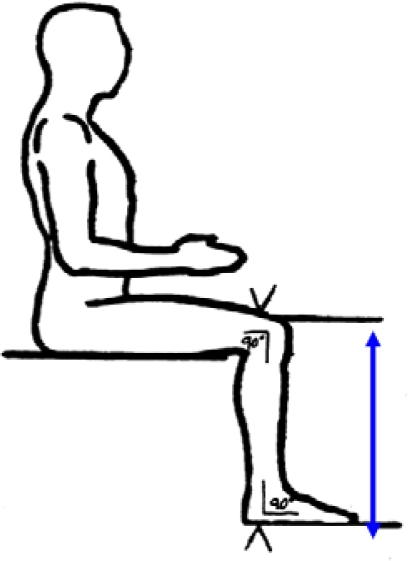
Knee height (from NHANES anthropometric manual).

The distance between the anterior surface of the thigh (above the condyles of the femur and about 4cm above the patella) and the floor (see figure 3).

### 3.5. Sitting Height Ratio (SHR)

**Figure 4.**
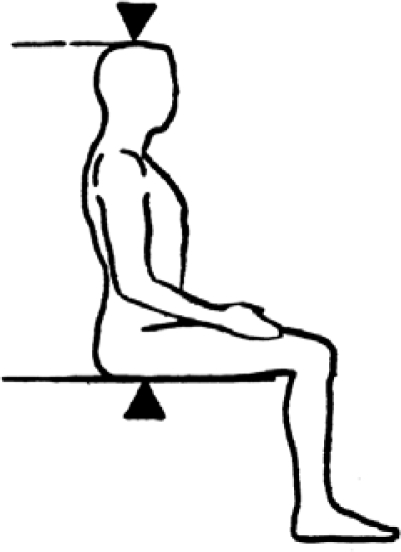
Sitting Height is measured from the vertex of the head to the seated buttocks (from NHANES anthropometric manual).

SHR is calculated as (Sitting Height / Height) × 100. It defines the percentage of total stature that is comprised by head and trunk (see figure 4 for details on sitting height [SH] measurement). The remaining portion of the body will be the length of the legs. The lower the SHR the relatively longer the legs are. SHR allows individuals with different heights to be compared in terms of the percentage of the body that is composed by the relative length of legs. Because it is SH dependent, this measure can be overestimated in individuals with high levels of gluteo-femoral fat, therefore underestimating the relative contribution of the lower limb to total stature [9]. There are international references [10] that allow the comparison of any values and the conversion of SHR raw data into percentiles and z-scores.

### 3.6. Relative Subischial Leg Length (RSLL)

RSLL is calculated as H-SH/H × 100. It defines the percentage of total stature that is comprised by the legs. The lower the RSLL the shorter the legs. There are no international reference values and it requires a harder computation of values of stature and sitting height.

### 3.7. Knee Height Ratio (KHR)

KHR is calculated as KH/Hx100. It defines the percentage of total stature that is comprised by the lower segment of the leg (tibia + foot height). The higher the KHR the longer the leg segment. There are no international reference values.

## 4. Evolutionary Background of Human Body Shape

**Figure 5.**
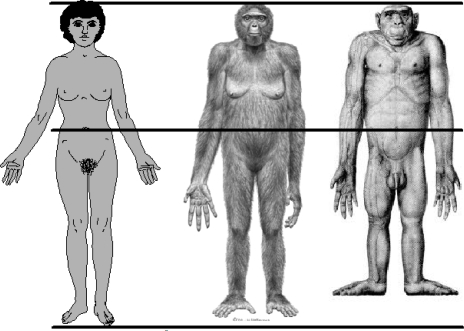
Approximate body proportions of *Homo sapiens, Ardipithecus ramidus* (4.4 MYA hominin, probable life appearance), and *Pan* troglodytes (chimpanzee). The figures are aligned at the crown of the head and the umbilicus to approximate a constant trunk length. Relative to trunk length, humans have the longest legs and shortest arms. Credits, *Homo sapiens*, SlideWrite Plus, 4.1, with authorization; *Ardipithecus ramidus, Science* 02 October 2009, ©J.H. Matternes, http://www.jay-matternes.com/; *Pan troglodytes*, Schultz, A. H. (1933). Die Körporproportionen der erwachsenen catarrhinen Primaten, mit spezieller Berüchsichtigung der Menschenaffen. *Anthropologischer Anzeiger* 10: 154–85 with permission of the publisher, http://www.schweizerbart.de.

The human species is distinguished from the non-human primates by several anatomical features. Among these are proportions of the arms and legs relative to total body length. The human difference is illustrated in Figure 5. In proportion to total body length, measured as stature, modern human adults have relatively long legs and short arms. Quantitative differences between adult humans, chimpanzees (*Pan troglodytes*), and bonobos (*Pan paniscus*) are given in Table 1. The combined values for the intermembral index and the humerofemoral index show that humans have leg bones averaging 34% longer then the non-human apes, relative to the length of arm bones. The primary reason for this is human bipedal locomotion, a behavior which evolved at least by 4.4 million years ago (MYA), as shown in the fossil hominin species *Ardipithecus ramidus*. Leg length must approximate 50 percent of total stature to achieve the biomechanical efficiency of the human striding bipedal gait. In modern humans this happens at the end of the childhood life history stage, which occurs at about 7.0 years of age [11]. By adulthood, human species-specific body proportions allow for not only the bipedal striding gait, but also—as has been observed, experimentally tested, or speculatively proposed—for technological manipulation [12], more efficient thermoregulation in a tropical savannah environment [13–16], the freeing of the hands for carrying objects and infants [17], for long distance running [18], and for gesticulation, communication, language, and social-emotional contact [19].

**Figure 6.**
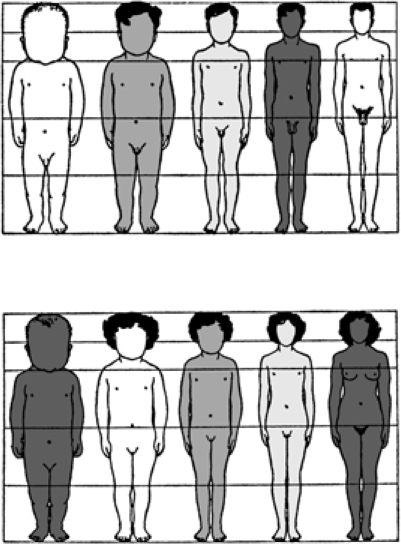
Changes in body proportion during human growth after birth. Ages for each profile are, from left to right, newborn, 2 years, 6 years, 12 years, 25 years. The hair style and shading of the cartoon silhouettes are for artistic purposes and is not meant to imply any ethnic, eco-geographical, or “racial” phenotypic characteristics of the human species [provided courtesy of Dr. J. V. Basmajian].

**Figure 7.**
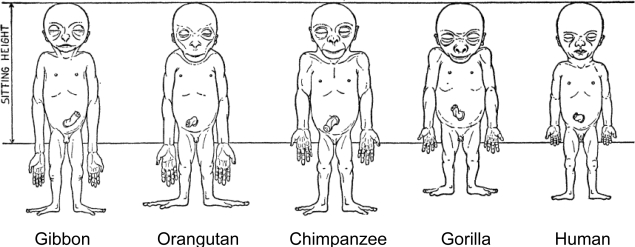
Schultz’s sketches of the body proportions of hominoid fetuses. The original legend for this figure states, “All the figures have the same sitting height. The human fetus is the 4^th^ month, the gorilla and the gibbon fetus correspond in development to the human fetus, but the chimpanzee and the orang fetus are slightly more advanced in their growth” [23, p. 465–466, accessed from http://www.jstor.org/stable/2808286].

Human adult body proportions are brought about by differential growth of the body segments [21]. At birth, head length is approximately one quarter of total body length, while at 25 years of age the head is only approximately one-eighth of the total length. There are also proportional changes in the length of the limbs, which become longer relative to total body length during the years of growth [22]. The cartoons of Figure 6 shows the typical changes that take place in people from birth to age 25 years. Human beings follow a cephalocaudal gradient of growth and development, the pattern common to most mammals. There are, however, some species-specific features of human body plan development. In a classic 1926 article, Schultz [23] published his sketches of the body proportions of hominoid fetuses, reproduced here as Figure 7. The human fetus “of the 4^th^ month” has relatively shorter legs than the chimpanzee, orangutan or gibbon. This assumes that Schultz’s estimates of development for the non-human apes are correct (see Figure 7 legend). Another difference, not noted by Schultz, in proportion is the size of the cranium relative to the face, which is larger in the human fetus than in the chimpanzee, orangutan or gibbon.

This human pattern of change in body proportions during gestation to birth and then to adulthood may be explained, in part, by the evolution of bipedalism interacting with the evolution of a large and complex brain. Apes have a pattern of brain growth that is rapid before birth and relatively slower after birth. Humans have rapid brain growth both before and after birth [24,25]. Human newborns are bigger brained than any of the apes, although not so much bigger in terms of brain-body mass ratio (Table 2). The human-ape differences in brain mass and brain/body mass ratio are much larger at adulthood, and much of these differences are, in fact, achieved by 6.9 years of age[11,25]. More than brain mass, it is brain metabolic activity that is, perhaps, the crucial difference. The human newborn uses 87% of its resting metabolic rate (RMR) for brain growth and function. By the age of 5 years, the percent RMR usage is still high at 44%, whereas in the adult human, the figure is between 20 and 25% of RMR. At comparable stages of development, the RMR values for the chimpanzee are about 45, 20, and 9% respectively [26]. With such high metabolic demands from its brain, the human infant and child may well have been naturally selected to make trade-offs in the allocation of limited nutrients, oxygen, and other resources required to grow the brain *versus* other body parts. Trade-offs between the growth, development and maturation of body parts are common across the diversity of animal and plant life histories [27–29], including the human species [30,31]. From this perspective, the ultimate level reason that human leg growth is delayed during fetal and infant development is that it allows for rapid growth of the brain.

The proximate level controls of the trade-offs in the growth of body segments and organs are not well known. Genetic, hormonal and nutrient supply factors are likely to be involved. In a review of bone growth biology Rauch [32, p. 194] states, “Bone growth in length is primarily achieved through the action of chondrocytes in the proliferative and hypertrophic zones of the growth plate. Longitudinal growth is controlled by systemic, local paracrine and local mechanical factors. With regard to the latter, a feedback mechanism must exist which ensures that bone growth proceeds in the direction of the predominant mechanical forces. How this works is unknown at present.” It is known that the length of the proliferative columns in the growth plate correlates with the length of limbs, “…a species with long legs and short arms has longer columns at the knees and shorter at the elbows than an oppositly proportioned species” [33, p. 21].

**Figure 8.**
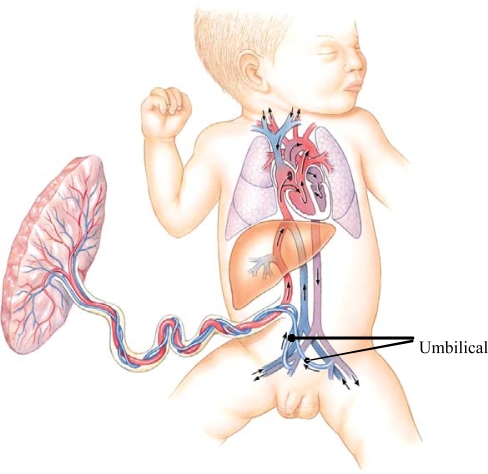
Human fetal circulation, adapted from [39] The relative amount of oxygen in the fetal blood is greatest in the upper thorax, neck and head; indicated by the red color of the vessels ascending from the heart. Blood flowing to the abdomen and legs is less well oxygenated; indicated by the violet color of the vessels descending from the heart.

Quantitative trait locus (QTL) mapping of laboratory mice has identified genomic regions associated with phenotypic differences in length of the femur, tibia, humerus and ulna [34]. Changes in genomic growth regulation, such as Hox expression patterns are known to be associated with growth of primate forearm segments [35]. Changes in the sensitivity of bone growth plates to growth promoting and inhibiting factors at different times during development, and at different sites of the skeleton, are also known to be responsible for differential growth of body segments [36,37]. A further speculation is that blood circulation of the fetus may contribute to the brain-leg growth trade-off. Blood in the fetal ascending aorta has higher oxygen saturation than does the blood descending to the common iliac artery (Figure 8). Additionally, the umbilical arteries carry some of the blood descending toward the leg back to the placenta. This pattern of fetal circulation is common to most mammals and is likely to be evolutionarily ancient. Combined with the more recently evolved metabolic demands of the human fetal brain, the ancient circulatory pattern may leave the human fetal legs with reduced supply of oxygen and nutrients, further slowing leg growth and development compared with more cephalic regions of the body. We can find no experimental support for this proposal. There is human clinical case study evidence that increased blood flow to the limbs is associated with greater amount of growth [38].

## 5. Size and Shape in Living Humans

**Figure 9.**
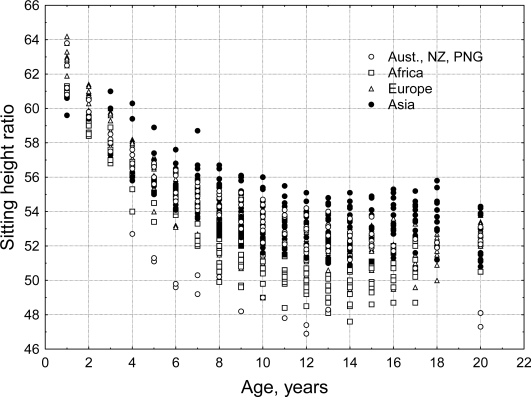
Sitting height ratio by age for the four geographic groups defined by Eveleth and Tanner [45,46]. Age 20 includes data for adults over the age of 18 years. A larger SHR indicates relatively shorter legs for total stature (authors’ original figure).

The general pattern of human body shape development is a species-specific characteristic. Historical artwork, sculpture and anatomical drawings from Renaissance Europe [40,41] and pre-Columbian Mexico[42] show fundamental commonalities in the depiction of body shape of late term fetuses, newborns and infants. Discrete populations of living humans, however, present a diversity of body sizes and shapes. Mean stature for populations of adults varies from minimum values for the Efe Pygmies of Africa at 144.9 cm for men and 136.1 cm for women [43] to the maximum values for the Dutch of Europe at 184.0 cm for men and 170.6 cm for women [44]. There are also biologically and statistically significant variation between human populations in body shape. Eveleth and Tanner [45,46] published data for body proportions and leg length, estimated via the sitting height ratio, from dozens of human populations, distributed across most geographic regions of the world (Figure 9). The sitting height ratio (SHR) is a commonly used measure of body proportion. Measured stature minus sitting height may also be used to estimate leg length but this measure does not standardize for total height making it difficult to compare individuals with different statures. Mean SHR for populations of adults varies from minimum values, *i.e.*, relatively longest legs, for Australian Aborigines (SHR = 47.3 for men and 48.1 for women) to the maximum SHR values, *i.e.*, relatively shortest legs, for Guatemala Maya men and Peruvian women (SHR = 54.6 and 55.8).

Making sense of these world-wide comparisons is difficult because of the differences in lifestyle, environment, and genomics. Two well-known ecogeographic principles, Bergmann’s and Allen’s Rules, are often cited as primary causes for the global patterns of human body shape variation. Bergmann [47], in 1847, observed that closely related mammalian species, such as bears, have greater body mass in colder climates. Allen [48] added in 1,877 that the limbs and tails of such species tend to be shorter in cold climates and longer in warmer environments. Large body mass and relatively short extremities increase the ratio of volume-to-surface area and provide for a body shape that maximizes metabolic heat retention in a mammal. Conversely, in warmer temperatures, relative long extremities increases surface areas relative to volume and allows for greater heat loss. It has been shown experimentally that mice and other non-human mammals raised in warmer temperature experience greater bone tissue growth and longer limb bones [49]. The usual explanation for this is greater vascularization, allowing for greater oxygen and nutrient perfusion. Recent experimental research shows, however, that even in the absence of vasculature, *in vitro* culture of chondrocytes from mouse metatarsal bone show a positive correlation between environmental temperature with, “…greater proliferation and extracellular matrix volume…” [49, p. 19348].

Bergmann’s and Allen’s rules apply, to some extent, to the human species. In 1953, Roberts [50] published an analysis showing a significant relationship between body mass and latitude for human beings, with groups of people living at higher latitudes having greater body mass than those living closer to the equator. Twenty-five years later, Roberts [51] updated and re-affirmed these findings. Other research shows that people living in colder regions also tend to have shorter limbs relative to total stature, compared with groups of people living in warmer regions [15,45,46].

These climate relationships, however, are not perfect. A re-analysis of the Roberts’ data by Katzmarzyk and Leonard [52] modifies the importance of climate as the primary molder of human body shape. Katzmarzyk and Leonard analyze the sitting height ratio of 165 human groups studied between 1960 and 1996. All of the human data analyzed by Roberts were collected prior to 1953. Katzmarzyk and Leonard show that the more recently studied groups still follow the ecological principles of body shape, but that the association with climate has been attenuated since Robert’s study. The slopes of the best fitting linear regression lines for the relation of mean annual temperature to sitting height ratio are half those reported by Roberts. Katzmarzyk and Leonard (p. 483) state that “...although climatic factors continue to be significant correlates of world-wide variation in human body size and morphology, differential changes in nutrition among tropical, developing world populations have moderated their influence.” The authors define the nutritional changes as modifications in diet and lifestyle, especially the introduction of western foods and behaviors. They point out that, “…climate may shape morphology through its influence on food availability and nutrition [meaning that] linear builds of tropical populations are the consequence of nutritional [factors] rather than thermal stress…” (pp. 491–492). In this case, during the years of growth and development more or less total food intake, more or less of any essential nutrient, more or less physical activity (and the type of activity) could influence body shape. Guatemala Maya, for example, consume only approximately 80% of the total energy needed for healthy growth, and 20.4% are also iodine deficient [53]. Iodine deficiency during infancy and childhood results in reduced leg length, especially the distal femur, the tibia and the foot [54]. Maya children and adults spend considerable time and energy at heavy labor [55], which diverts available energy in the diet away from growth. This nutrition and lifestyle combination is known to reduce total stature and leg length [56].

The body shape of people may have a genetic basis, especially for human groups who have resided in the same environment for many generations. A comparison of stature and body proportion between blacks (African-Americans) and whites (European-Americans) in the United States provides an example of genome-environment interactions and their affect on growth [57]. Published data from the first National Health and Nutrition Examination Survey (NHANES I) of the United States, gathered anthropometric data on a nationally representative sample of blacks and whites aged 18 to 74 years. When the data are adjusted for differences between the two ethnic groups in income, education, urban or rural residence, and age, there is no significant difference in average height between black and white men. Nor is there a significant difference in average height between black and white women.

Although white and black adults in the United States have the same average stature, when education, income and other variables are controlled, the body proportions of the two groups are different. Krogman [58] found that for the same height, blacks living in Philadelphia, USA had shorter trunks and longer extremities than whites, especially the lower leg and forearm. Hamill *et al*. [59] found that this was also true for a national sample of black and white youths 12 to 17 years old, and it is the case for adults 20−49 years old measured for the NHANES III survey, 1988–1994 [9]. A genomic contribution to the body proportion differences between blacks and whites seems likely, as the blacks tend to have more sub-Sahara African genomic origins than the whites.

Few if any specific genes for human body proportions are known. In a statistical pedigree analysis of two human samples, Livshits *et al*. [60] estimate that between 40% and 75% of inter-individual variation in the body proportions they studied (adjusted for age and sex) are attributable to “genetic effects”. These may be better described as familial effects because the authors analyzed families and also because they found significant common environmental effects for siblings as well as significant sex by age interactions. The range of the sources of variation in the analysis makes it difficult to compute simple genetic variance.

Even if specific genotypes are discovered, their direct contribution to normal ethnic (so-called “racial”) variation in human body shape may be relatively small. At 40 weeks gestation, fetuses identified as African-Americans have, on average, relatively longer legs than fetuses identified as European-Americans [23]. But the difference, as measured by (total length/crown-rump length) is less than 1%. In an analysis of the data shown in Figure 9, Bogin *et al*. [61] estimated the contribution of geographic origin to the variance in the SHR to be 0.04, which accords well with genomic estimates for variation in total stature of 0.04–0.06 [2]. Forensic anthropologists and physicians in the United States have often used “race-specific” body proportions, to ascribe an African-American or European/Asian-American ethnicity to a skeleton [63,64]. Feldesman and Fountain [65] tested the utility of the femur length/stature ratio to correctly identify 798 femur/stature pairs of skeletons of known ethnicity. They found that, “…the “Black” femur/stature ratio is statistically significantly different from those of “Whites” and “Asians” [p. 207]. Discriminant function and cluster analysis shows, however, that coherence to groups defined by geographic origin is poor, with results barely better than chance. Using “race-specific” body proportions to identify unknown skeletons would result in a high number of incorrect attributions of ethnicity.

A more promising approach to understanding the control of human body proportions comes from genomic research. *Hox* genes and homeobox sequences, and a growing number of growth and signaling factors, are known to regulate the growth of body segments [66], and these genes are shared across all taxa. There is observational and experimental evidence that Hoxd expression is linked with forearm, hand, and digit length differences in the apes [35]. The short stature homeobox-containing gene (SHOX) is another genomic region that may be relevant to human body proportions. “SHOX, located on the distal ends of the X and Y chromosomes, encodes a homeodomain transcription factor responsible for a significant proportion of long-bone growth [67]. Turner syndrome (45, XO karyotype) results in approximately 20 cm deficit in stature. Some studies find that legs are disproportionately affected [68,69], but other studies find no disproportion [70]. More specific candidate genes for body shape are known from some non-human mammals [71,72] and in insects [27].

Another very active area of research is epigenetic regulation of body growth [73]. Epigenome effects may act through a number of genome (e.g., DNA methylation and histone modification), proteome (e.g., micro-RNA regulation of gene expression), and environment (e.g., climate, diet and physical activity) interactions and may well play the major role in determination of human size and shape.

## 6. Developmental Plasticity

Plasticity refers to the concept that the development of the phenotype of an organism is responsive to variations in the quality and quantity of environmental factors required for life [74]. We employ this concept here to mean that during the years of growth and development, humans can grow more or less of various tissues and come to be adults of various sizes and shapes. As adults these sizes and shapes are largely fixed, especially for total stature and the length of body segments. Human growth is highly plastic during the years of growth and development, responding to the overall quality of living conditions [11]. From the perspective of developmental plasticity, leg length, both in terms of absolute size and relative to total stature, is an indicator of the quality of the environment for growth during infancy, childhood and the juvenile years of development.

The reason for this is the general principle that those body parts growing the fastest will be most affected by a shortage of nutrients, infection, parasites, physical or emotional trauma, and other adverse conditions. The cephalo-caudal principle of growth as applied to the human species means that the legs, especially the tibia, are growing faster relative to other body segments from birth to age 7 years. Relatively short LL in adolescents and adults, therefore, is likely to be due to adversity during infancy and childhood leading to competition between body segments, such as trunk *versus* limbs and between organs and limbs. In the simplest case, such competition may be for the limited nutrients available during growth [31,56,61]. More complex explanations for competition relate to aspects of the thrifty phenotype hypothesis [75,76], the intergenerational influences hypothesis [77,78], the fetal programming hypothesis [79], and the predictive adaptive response hypothesis [80,81]. Discussion of these hypotheses is beyond the scope of this review [see reference 31, and other articles in the same issue, for such discussion], but in essence each of these hypotheses predicts that the vital organs of the head, thorax, and abdomen of the body will be protected from adversity at the expense of the less vital tissues of the limbs.

## 7. The Use of Leg Length in Human Biology and Environmental Epidemiology

Leitch [82] was the first medical researcher to propose that a ratio of LL to total stature could be a good indicator of the early life nutritional history and general health of an individual. Leitch (p. 145) wrote, ‘. . . it would be expected on general principles that children continuously underfed would grow into underdeveloped adults. . .with normal or nearly normal size head, moderately retarded trunk and relatively short legs.’ Reviewing the literature available at the time (pre-1950), Leitch found that improved nutrition during infancy and childhood did result in a greater increase in LL than in total height or weight. One of the critical studies in her review is the Carnegie U.K. Dietary and Clinical Survey, which recorded height, weight and iliac height (IH). When the participants were grouped by age and family expenditure on food, it was found that IH, ‘…was consistently better than total height for indicating [food] expenditure group’ (p. 213). Leitch also reported that longer-legged children were also less susceptible to bronchitis, which was then a scourge of poorly fed children.

Leitch was careful to state that leg length *per se* is not a direct cause of better or worse health and that children and adults with relatively short legs may be quite healthy. She viewed greater leg length as a correlate of an improved constitution. This view anticipates current biomedical research on the development of somatic and cognitive reserve capacity [83–85] in relation to health and rate of senescence. The Reserve Capacity Hypothesis posits that during human growth and development the somatic and cognitive systems usually “overshoot” their minimally necessary capacity for sustaining life of the individual. By overshooting this necessary capacity an individual has reserve capacity which may be channeled into greater growth, better health, more successful reproduction, social and economic success, and slower rates of senescence. Leg length relative to total stature may be one indicator of overall reserve capacity of a person or a group of people.

### 7.1. Leg Length and Human Environmental Health

Many studies support Leitch’s findings and hypothesis [86–99]. In the past 10 years the number of publications on the relationship of leg length to human health has increased at a rapid rate. A systematic review of these studies is not provided here, instead we sample some of the literature to provide an overview of research.

Table 3 summarizes several recent studies that show how leg length and body proportion ratios are powerful indicators of the quality of the environment and of the plasticity of the human body. The table provides only a few studies, of which there are dozens. What is important to note is that regardless of the specific leg measure taken, longer LL is associated with better environments, better nutrition, higher SES, and better general health, overall.

Poor childhood health, insufficient diet, adverse family circumstances and maternal smoking during pregnancy are each known to reduce leg length [104,111–114]. Frisancho *et al.* [107] emphasize the environmental effects in a study that finds that leg length of Mexican-Americans aged 2–17 years old is significantly associated with socioeconomic status of their families. In that study, individuals from better-off families have significantly longer legs, but equal trunk length, when compared with boys and girls from poorer families. Dangour [115] reports similar findings for two tribes of Amerindian children living in Guyana. The tribes are both of low socioeconomic status, but differ markedly in the quality of their living conditions. Children in the tribe with better living conditions are taller than their age-mates in the other tribe. The difference in stature is due almost entirely to differences in leg length, as there are no significant differences in sitting height between the tribes.

Our own studies are of Maya families from Guatemala migrated to the United States from the late 1970s to the early 1990s [31,56,116–119]. In Guatemala the Maya are subjected to chronic adversity in the form of poor nutrition, heavy workloads, contaminated drinking water, infectious disease, limited education opportunities, and state supported violence. In the United States the Maya tend to occupy low socioeconomic status (SES) and work at physically demanding jobs, but benefit from safe drinking water, copious food availability, public education, health care, and relative safety. Births to Maya immigrant women created a sizable number of Maya-American children. We measured the height and sitting height of 5 to 12 years-old children (n = 431) in 1999 and 2000, and from these measurements leg length is estimated and the sitting height ratio is calculated. We compare these data with a sample of Maya children of the same ages living in Guatemala measured in 1998 (n = 1347). Maya-American children are currently 11.54 cm taller and 6.83 cm longer-legged, on average, than Maya children living in Guatemala. The values indicate that about 60% of the increase in stature is due to longer legs. Consequently, the Maya-Americans have a significantly lower average sitting height ratio than do the Maya in Guatemala.

### 7.2. Leg Length and Risk for Morbidity and Mortality

Decomposing stature into its major components is proving to be a useful strategy to assess the antecedents of disease, morbidity and death in adulthood [120–123]. Human leg length, however it is measured, trunk length and their proportions (e.g., relative leg length or the sitting height ratio [sitting height/stature]) are associated with epidemiological risk several diseases and syndromes. Relatively shorter legs and shorter stature due to relatively shorter legs may increase the risk for overweight (fatness), coronary heart disease and diabetes [103,112,122–125]. These same proportions are associated with liver dysfunction (increased levels of the liver enzymes alanine aminotransferase, gamma-glutamyltransferase, aspartate transaminase and alkaline phosphatase) [126]. In a systematic review of the literature prior to 2001, Gunnell *et al.* [127] find that some cancers, such as prostate and testicular cancer, premenopausal breast cancer, endometrial cancer and colorectal cancer, are statistically more likely in adults with greater stature and relatively longer legs. These authors report that the positive relationship between leg length and risk for these cancers may be due to the effects of insulin-like growth factor 1 (IGF-1). Gunnell and colleagues write that, “…raised levels of IGF-I are associated with increased risks of prostate, breast, and colorectal cancers. The most potent cell survival factor controlling apoptosis is insulin-like growth factor I (IGF-I). Raised levels of IGF-I and reduced levels of its main binding protein, insulin-like growth factor (IGF)-binding protein 3, may diminish this defense against a range of cancers” [127. p. 313, citations in the original omitted]. Since 2001 several more reports of a relationship between IGF-1, IGF-1 receptors, and cancer risk have been published (e.g., [128,129]) as well as associations between IGF-2 and IGF-2 receptors and cancer risk [130]. A search of PubMed.gov using the terms “cancer, IGF” leads to 4123 articles published in the past 10 years. This is an active area of research, often reporting contradictory findings, but not reviewed further in the present article.

There are complications in the relationship between LL, health, SES, and better environments for growth. One such complication is noted by Schooling *et al*. [131,132] in an analysis of a cross-sectional sample from of 9998 Chinese people aged at least 50 years old and measured in 2005–2006. SH and H were measured and LL estimated as H-SH. The growth environment for the 50+ year old adults was estimated via a questionnaire asking about own education, father’s occupation, parental literacy, and parental possessions. The authors find that leg length and height, but not sitting height, vary with some childhood conditions. Participants with two literate parents who owned more possessions have longer legs. Unexpectedly, the participants’ education level and their father’s occupation have no effect on height or leg length. Higher scores for these variables do associate with an earlier age at menarche for women participants. The authors explain that earlier menarche for girls, and earlier puberty for boys, will terminate growth at an earlier age. This may explain why higher SES of the participants and their parents, as measured by education and father’s occupation, did not associate with longer LL. That parental literacy and possessions did associate with LL indicates that researchers must focus on factors that are socially and historically relevant to the population under study, rather than a generic measure of SES.

Another complication is noted by Padez *et al*. [133], who analyzed the growth status of Mozambique adolescents. The sample comprised 690 boys and 727 girls, aged between 9 and 17 years, from Maputo, the capital city. The sample is divided between those living in the center of Maputo (higher SES) and those living in the slums on the periphery of the city. Height, weight, and sitting height were measured and the sitting height ratio was calculated. The hypothesis that relative leg length is more sensitive than total stature as an indicator of environmental quality is not uniformly confirmed. Overall, mean stature is greater for the centre group than the slum group, but relative leg length as measured by the sitting height ratio does not differ. Compared with African-American references (NHANES II), all center girls, 9- to 14-year-old slum girls, all slum boys, and the oldest centre boys show relatively shorter legs. These findings show that within the Mozambique sample, relative leg length is not sensitive enough to distinguish the quality of the living environment. A reason for this is that Mozambique was a colony of Portugal until 1975. Civil unrest and warfare characterized the late Colonial period and the post-independence period until a peace settlement was concluded in 1992. It is possible that all socioeconomic status groups exposed to the civil war within the country suffered sufficiently to reduce relative leg length compared with the better-off African-American reference sample.

## 8. Leg Length and Beauty

“The legs, besides being a very important functional unit, are also an important sexual attraction in themselves, and in all cultures they have a preponderant place in the concept of beauty” [134, p. 505]. A concern with body proportion has deep roots in European history. Building on the work of Vitruvius, a first century B.C. Roman architect and writer, Leonardo da Vinci (b1452–d1519) developed canons, or rules, for drawing human proportions. According to these canons, human body height is to be the length of eight heads, with an additional one-quarter head for neck length. Leg length is to be four head lengths. Leonardo’s “Vitruvian Man” (c. 1487) is the iconic illustration of the canons. Albrecht Dürer (b1471–d1528), a German artist, devised technology to draw both the canonical forms and many variations as observed in nature. With his geometric methods, Dürer could draw any manner of human variation in size or proportion. He applied his method to drawings of men, women, children and infants. Including women and children in this type of methodological work was an innovation, as most artists followed the teachings of Cennino Cennini (c. b1400) who wrote that women do “... not have any set proportion” [40, p. 202]. Children, it seems, were too inconsequential for Cennini to even mention!

After the year 1600, the post-Renaissance painters begin to depict children with normal proportions and also with growth pathologies. The Flemish artist Van Dyck depicts three normal children in the painting “The Children of Charles I” (1635). The painting “The Maids of Honor” by Diego Velazquez (1656) depicts a normal child, a woman with achondroplastic dwarfism (normal sized head and trunk with short arms and legs) and a man with growth-hormone deficiency dwarfism (proportionate reduction in size of all body parts). At the time of these paintings, of course, the biological control of normal and pathological growth in size and proportion was not known.

Edmund Burke, the British statesman and philosopher, published in 1756 the essay, “The Philosophical Inquiry into the Origin of Our Ideas on the Sublime and Beautiful”. One part of this essay is sub-titled, ‘Proportion not the Cause of Beauty in the Human Species’. Burke argued that people with body proportions outside the canon of Leonardo might still be considered beautiful. He held the human leg to be especially handsome, ‘I believe nobody will think the form of a man’s leg so well adapted to running, as those of a horse, a dog, a deer, and several other creatures; at least they have not that appearance: yet, I believe, a well-fashioned human leg will be allowed to far exceed all these in beauty’. One is left to wonder which human legs are ‘well-fashioned’. Perhaps Burke meant those that are relatively straight and long—contraindicating rickets, suggesting good health and nutrition in childhood, and predicting fecundity in adult women.

The intersection of biomedical and aesthetic concern with the beauty of human leg is still strong today. The quote from Cuenca-Guerra and colleagues [134] that open this section is from an article on the surgical use of calf implants to enhance leg attractiveness. There is a burgeoning literature on the scientific analysis of beauty and the medical means to enhance it, much of which focuses on body proportion and leg length (e.g., [135–137]).

## 9. Conclusion

Cosmetic surgery, elevated heels on shoes, and other clever styles of clothing can make legs more attractive, but these techniques do not overcome the fundamental linkages between leg length and human health. A broad review of the literature indicates that there is good evidence that adults with skeletal disproportions, especially high SHR (short legs), are at greater risk for coronary heart disease (CHD) via hypercholesterolaemia, impaired glucose and insulin regulation, increased pulse pressure and systolic blood pressure, and higher fibrinogen levels [103]. Some cancers are associated with relatively long legs.

Prenatal and postnatal undernutrition and disease account for relatively short legs in adults, but still do not explain why they are at greater risk for disease and mortality at earlier ages than the longer-legged adults. An association between childhood stunting and adult overweight is becoming well known. A prospective 3 year study of stunted Brazilian boys and girls, 11–15 years old, finds that they gain more fat mass and less lean body mass compared with non-stunted peers [138]. Brazilian adult women with short stature and disproportionately short legs have high risk for obesity [139]. The reason for these associations with fatness seems to be tied to impaired fat oxidation in stunted children [140]. Fasting respiratory quotient (RQ = the ratio of the volume of carbon dioxide produced by an organism to the volume of oxygen consumed) is significantly higher, and hence fat oxidation is lower leading to greater body fat stores in the stunted group. Other contributors may be impairment of appetite control associated with early malnutrition and lower resting and postprandial energy expenditure [141].

Early life undernutrition and disease not only reduces leg length relative to total stature, but may also alter human physiology toward a phenotype with a deranged metabolism. Understanding the nature of metabolic impairments may provide entrée toward an explanation for the relationship between measures of leg length with risks for overweight/obesity, diabetes, hypertension, low bone density, CHD, other human pathologies, and premature mortality. Edmund Burke may have found relatively short legs to be capable of beauty, but the epidemiological evidence finds them to be a risk for health.

## Figures and Tables

**Table 1.** Long bone indices of Humans and Chimpanzees [20]. All indices are based on measurements of the maximum length of the long bones. Intermembral index = [(humerus + radius) × 100] / (femur + tibia), Humerofemoral index = (humerus × 100) / femur.

Species	Intermembral Index	Humerofemoral Index
Human (male)	69.7	71.4
Human (female)	68.5	69.8
Chimpanzee (male)	108.0	101.1
Chimpanzee (female)	109.4	102
Bonobo (male & female)	102.2	98.0

**Table 2.** Neonatal and adult brain weight and total body weight for the great apes and human beings. Adult body weight is the average of male and female weight. Data from [142].

	Neonatal mass (grams)			Adult mass (grams)		
Species	Brain	Body	Br/Bo ratio	Brain	Body	Br/Bo ratio
*Pongo* (orangutan)	170.3	1,728.0	0.10	413.3	53,000.0	0.008
*Pan* (chimpanzee)	128.0	1,756.0	0.07	410.3	36,350.0	0.011
*Gorilla*	227.0	2,110.0	0.11	505.9	126,500.0	0.004
*Homo sapiens*	384.0	3,300.0	0.12	1,250.0	44,000.0	0.284

**Table 3.** Summary of a few studies published since 2000 employing measures of leg length in relation to early life living conditions and health.

**Measure of “leg length”**	**Sample sizes**	**Sample**	**Results**	**Source**
**IH**	Total: 2,209 M: 1,062 F: 1,147	2–14 years Extracted from The Boyd Orr Survey. Children from 1343 working class families in England and Scotland, measured between 1937 and 1939	M&F: positive association with length of breastfeeding, decreasing numbers of children in the household and increasing household income. Overall, the individual components of stature mostly associated with childhood environment was leg length (measured as IH) and foot length (not in the scope of this entry).	[100]
	Total: 916 M: 376 F: 540	65+ years inhabitants of Kwangju, South Korea, assessed in 2003.	Shorter limb length is associated with markers of lower early-life socioeconomic status and is associated with dementia later in life, especially in women.	[101]
**SLL**	Total: 2,338 M: 1,040 F: 1,298	30–59 years (United Kingdom)	M&F: inverse association with systolic BP, diastolic BP, total cholesterol and fibrinogen. Direct association with FEV, FVC, BW, and BMI	[102]
	Total: 10,308 M: 6,895 F: 3,413	35–55 years (London)	M&F: Strong inverse association with pulse pressure and systolic BP. Strong positive association with lower total/HDL cholesterol ratio, triglycerides, and 2hr glucose M: Strong inverse association with total cholesterol. F: Strong inverse association with diastolic BP.	[103]
	Total: 3,262	Longitudinal study, births from 3–9 March 1946. 21 assessment occasions between birth and 53 years). MRC National Survey of Health & Development (United Kingdom)	M&F: Positive association with mother’s & father’s height, BW. SLL greater among individuals from non-manual social class and among individuals who were breastfed	[104]
	Total: 5,900	The 1958 British Birth Cohort. Participants assessed at birth and at ages 7, 11, 16, 23, 32, 42, and 45	Adult SLL associated with parental height, birth weight. Taller prepubertal stature is associated with higher SLL. Maternal smoking during pregnancy resulted in lower adult SLL. Overall, adult SLL is related to a greater extent than trunk length to early life factors and prepubertal height	[105]
**KH**	Total: 50 M: 27 F: 23	Infants grouped by gestation time at birth: <28 weeks, 28–31 weeks, 32–36 weeks, >36 weeks. Births occurred in 2004–2005, in the neonatal intensive care, Christchurch, New Zealand.	Changes in KH (using a kneemometer) correlate very well with changes in weight. If gain in weight is achieved, normal linear growth may be assumed. Because of this, kneemometry is not a useful addition to routine measurements of growth in the neonatal unit	[106]
**SHR**	Total: 2,985 M: 1,465 F: 1,520	2–17 years Mexican-Americans (NHANES III, USA)	M&F: Individuals with relatively shorter legs in proportion to total stature are poorer than longer “legged” individuals (poverty assessed by Poverty Income Ratio)	[107]
	Total: 1,472 M: 747 F: 707	6–13 years, Oaxaca, Southern Mexico Urban in 1972: Total:409, M:218, F:173 Rural in 1978: Total:363, M:179, F:184 Urban in 2000: Total:339, M:173, F:166 Rural in 2000: Total:361, M:177, F:184	Positive time trend in leg length from 1972 to 2000 both in rural and urban settings	[108]
	Total: 2003 M: 2003 F: 0	7–16 years. Two cross-sectional surveys among school aged boys from Kolkata, India. 1982–1983 (n = 816) 1999–2002 (n = 1187)	Positive time trend in relative leg length. Boys measured in 1999–2002 had relatively longer legs in proportion to total stature than their counterparts in 1983–1983.	[109]
	Total: 1995 M:977 F: 1018	5–12 years. Maya migrants to the USA in 1992 (n = 211), Maya migrants to the USA in 2000 (n = 431) and Maya in Guatemala in 1998 (n = 1353)	Leg length is a sensitive indicator of the quality of the environment. Maya children in the USA show relatively longer legs in proportion to stature than their counterparts in Guatemala. By 2000, Maya migrants to the USA were 11.54 cm taller and 6.83 cm longer-legged than Maya children in Guatemala.	[56]
**RSLL**	Total: 273	Intergenerational sample Parents’ generation: Total:165, M:80, F:85 Offspring generation: Total:108, M:49, F:59 From Auckland and Taipei	Is an effective marker of intergenerational changes	[110]
**KHR**	Total: 273	Intergenerational sample Parents’ generation: Total:165, M:80, F:85 Offspring generation: Total:108, M:49, F:59 From Auckland and Taipei	Is an effective marker of intergenerational changes. Lower leg growth, as represented by KHR is similar to changes in overall leg length in sensitivity to environmental change.	[110]
